# Supplementary material for: 3D imaging reveals changes in the neurovascular architecture of the murine calvarium with aging
Source: Bone Res. 2025 Feb 21;13:24. doi: 10.1038/s41413-025-00401-8 (PMC11845787; doi:10.1038/s41413-025-00401-8)
Supplement: Supplementary file 1 — Supplemental Materials [file 41413_2025_401_MOESM1_ESM.pdf]

Supplemental Figures

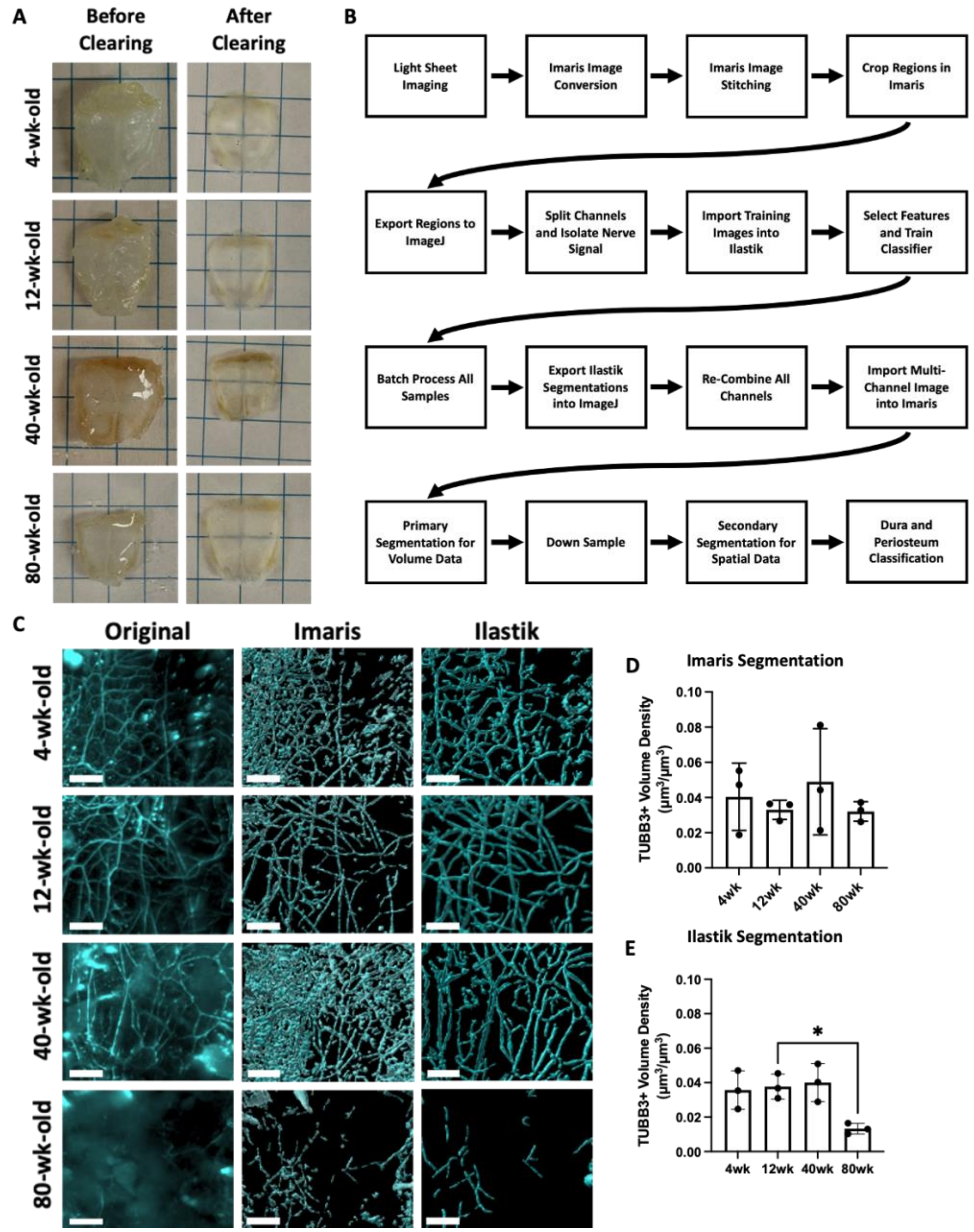

Supplemental Figure 1: A method for quantitative analysis of 3D nerve structures acquired with QLSM. A) 4wk, 12wk, 40wk, and 80wk calvaria before and after tissue clearing with 2’2-thiodiethanol. B) Workflow of quantitative nerve analysis beginning with lightsheet imaging, followed image processing with Imaris®, ImageJ®, and Ilastik®. C) Comparison of segmentation of nerves using Imaris® and Ilastik® relative to the original fluorescent images acquired with QLSM. Scale bar is 250 µm. D) Imaris®-derived volume fraction for TUBB3+ nerves in the frontal bone region. E) Ilastik®-derived volume fraction for TUBB3+ nerves in the frontal bone region. Data are mean ± SD. Statistics were performed with a two-way ANOVA with post-hoc Tukey HSD test. *$p<0.05$ where designated.

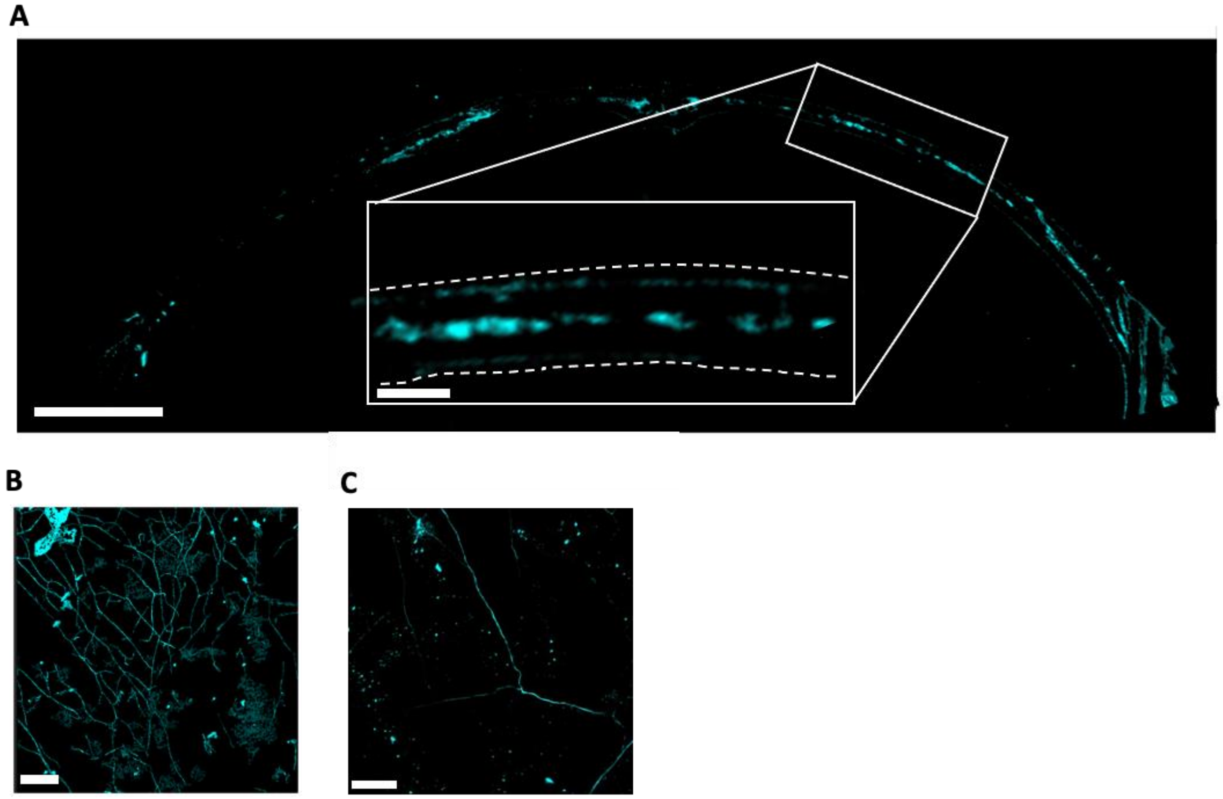

Supplemental Figure 2: Other methods for imaging calvarial nerves. A) 50 µm coronal cross section of TUBB3+ nerves from sectioned tissue. Scale bar is 1000 µm. Inset scale bar is 150 µm. B) Maximum intensity projection of 10x confocal image of whole mount calvaria tissue in the parietal bone. Scale bar is 200 µm. C) Maximum intensity projection of 20x confocal image of whole mount calvaria tissue in the parietal bone. Scale bar is 50 µm.

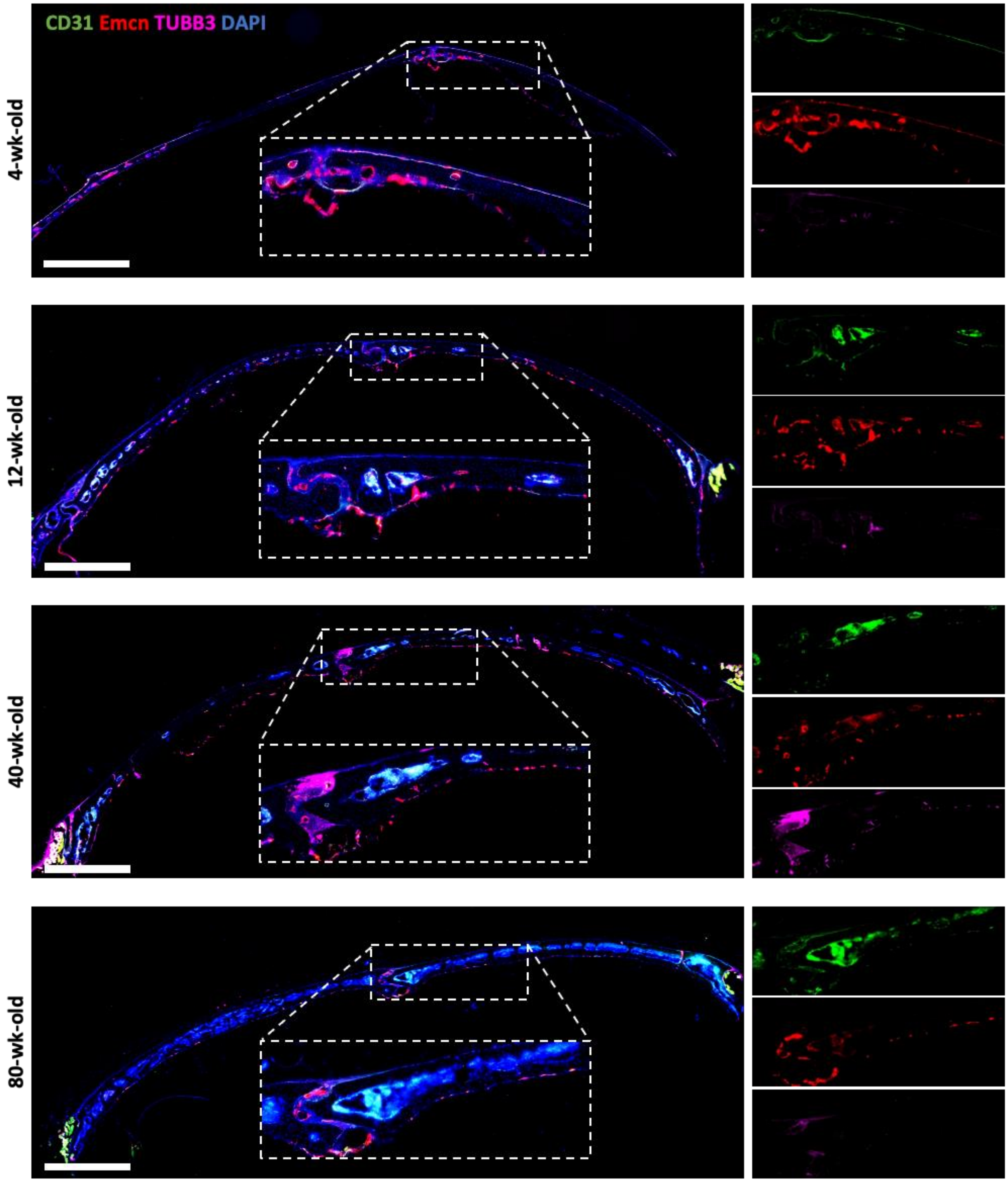

Supplemental Figure 3: Calvarial cross-sections with aging. 30 µm coronal cross section of TUBB3+ nerves from sectioned tissue in 4-wk-old, 12-wk-old, 40-wk-old, and 80-wk-old samples. Scale bar is 1000 µm.

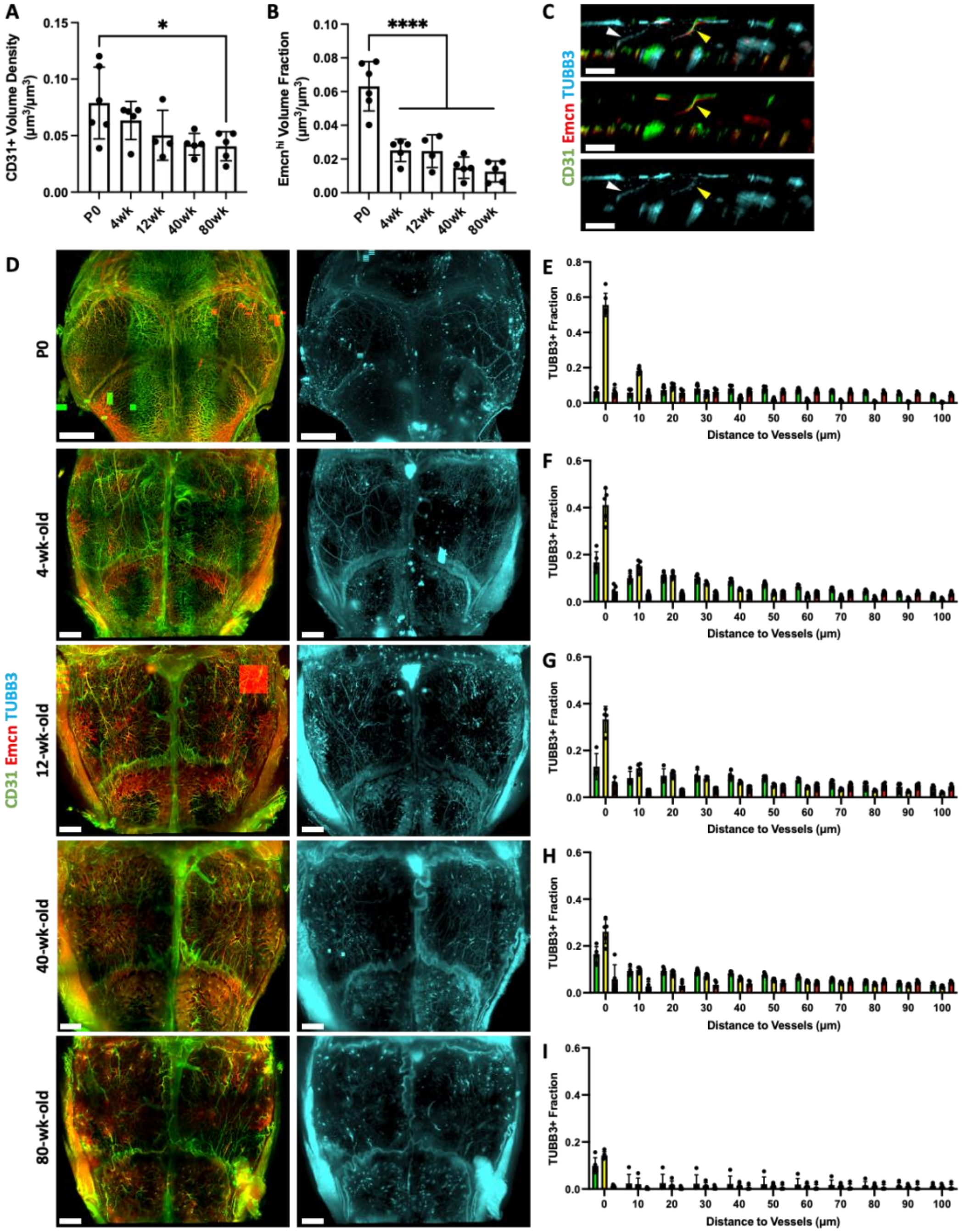

Supplemental Figure 4: Changes in TUBB3+ nerve interactions with vasculature during aging. A) Vessel volume calculations for CD31+ blood vessels for P0, 4wk, 12wk, 40wk, and 80wk calvaria. B) Vessel volume calculations for Encn+ blood vessles for P0, 4wk, 12wk, 40wk, and 80wk calvaria. C) 50 µm coronal cross section of TUBB3+ nerves and CD31+ and Emcn+ blood vessels. White arrowheads represent nerves and vessels in transcortical canals. Scale bar is 300 µm. D) Full calvaria MIP images for CD31+ and Emcn+ blood vessels and TUBB3+ nerves in P0, 4wk, 12wk, 40wk, and 80wk mice. Scale bar is 1000 µm. E-I) Spatial association histograms from TUBB3+ nerve association to $CD31^{hi}Emcn^{-}$ (green), $CD31^{hi}Emcn^{hi}$ (yellow), and $CD31^{lo}Emcn^{hi}$ (red) blood vessels in E) P0, F) 4wk, G) 12wk, H) 40wk, and I) 80wk mice.

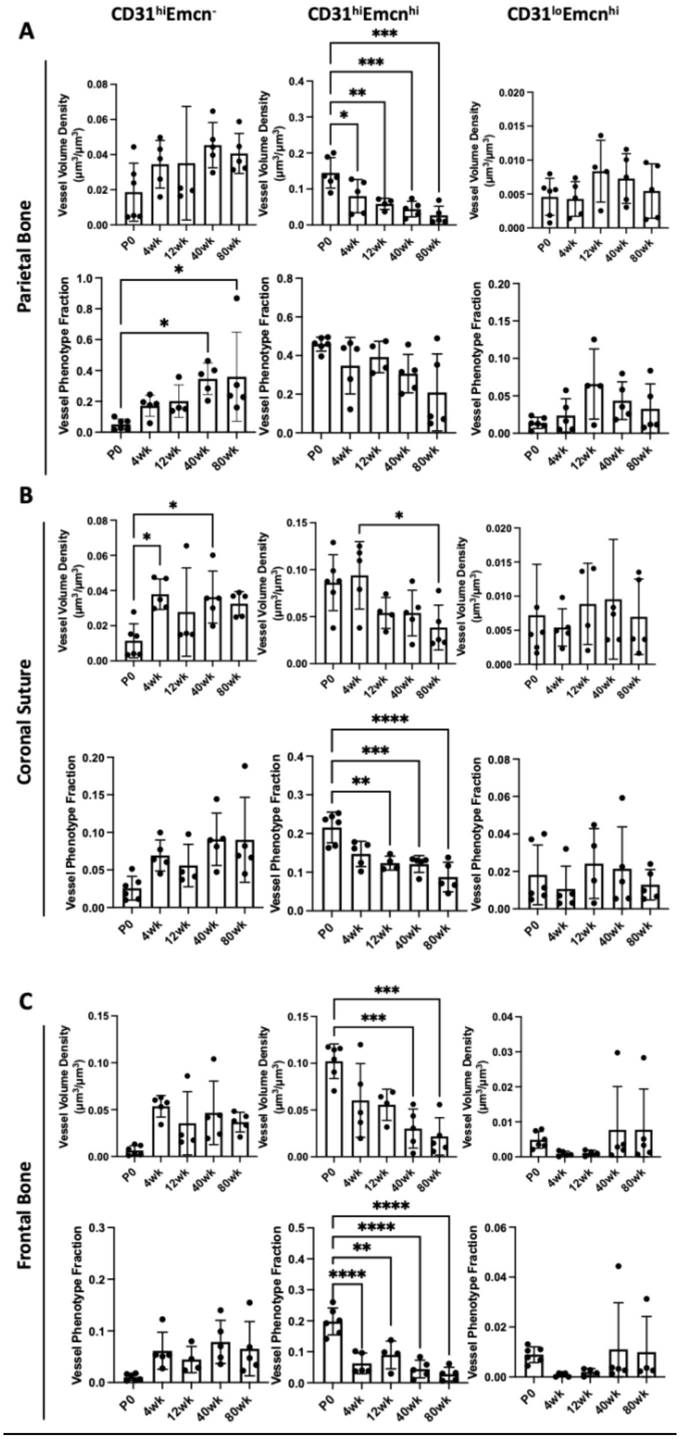

Supplemental Figure 5: Regional changes in vessel phenotype distributions over the mouse lifespan. A) Vessel phenotype volume density and fraction for CD31^{hi}Emcn^{-}, CD31^{hi}Emcn^{hi}, CD31^{lo}Emcn^{hi} blood vessels in the parietal bone for P0, 4wk, 12wk, 40wk, and 80wk calvaria. B) Vessel phenotype volume density and fraction for CD31^{hi}Emcn^{-}, CD31^{hi}Emcn^{hi}, CD31^{lo}Emcn^{hi} blood vessels in the coronal suture region for P0, 4wk, 12wk, 40wk, and 80wk calvaria. C) Vessel phenotype volume density and fraction for CD31^{hi}Emcn^{-}, CD31^{hi}Emcn^{hi}, CD31^{lo}Emcn^{hi} blood vessels in the frontal bone for P0, 4wk, 12wk, 40wk, and 80wk calvaria.

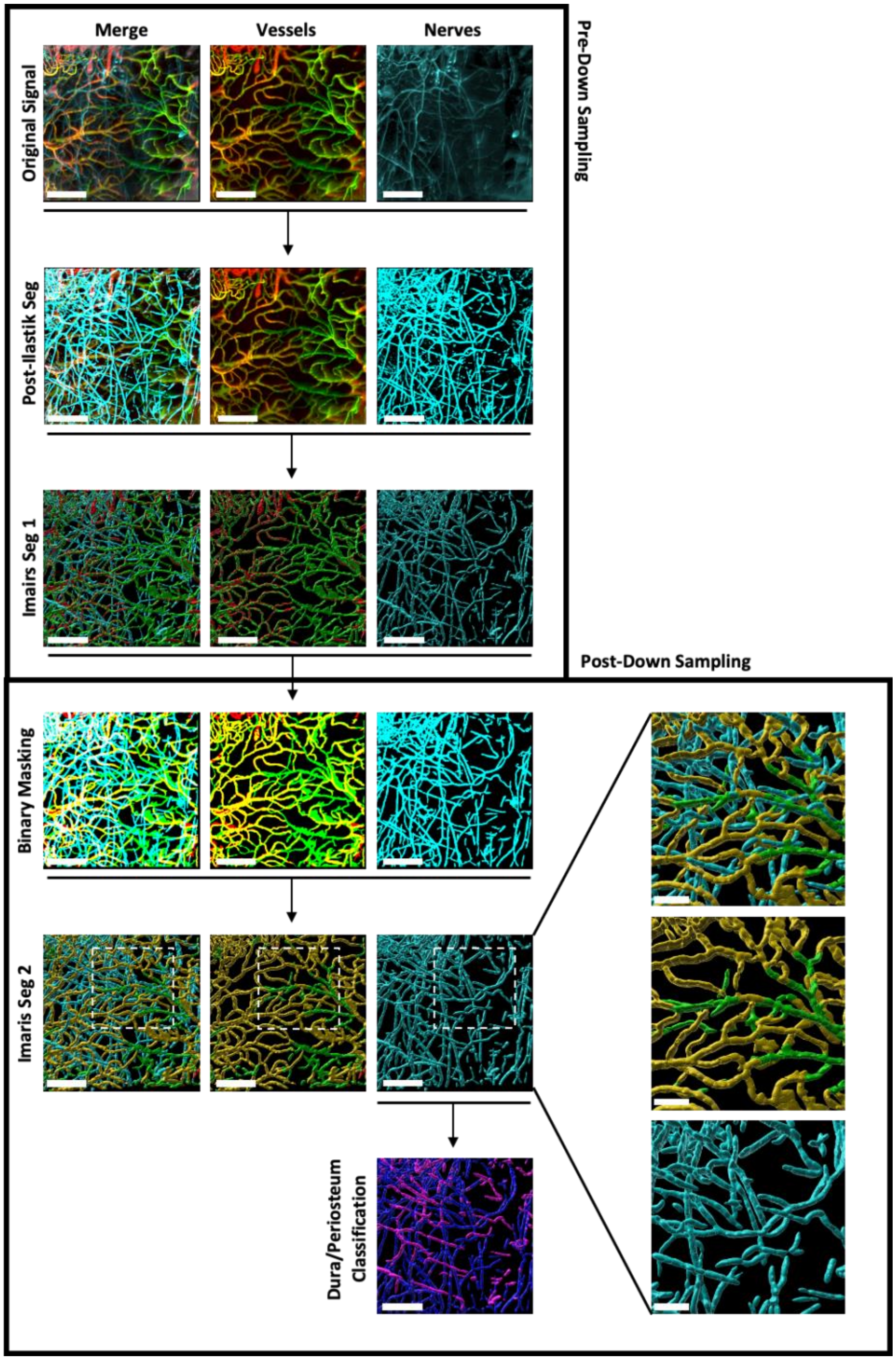

Supplemental Figure 6: Workflow for image quantification with Imaris®. Prior to spatial down-sampling and post-Ilastik® nerve segmentation, nerves and blood vessels undergo an initial segmentation with Imaris® to generate individual channel volume data. Following down-sampling, initial Imaris® segmentations are used to generate binary masks for each channel. Next, a secondary segmentation is conducted with Imaris® to generate vessel phenotype classifications, spatial association analysis, and dura/periosteum analysis. The secondary segmentation splits surfaces into 10 µm sections for subsequent analysis (zoomed region). Scale bars are 300 µm. Zoomed region scale bars are 150 µm.

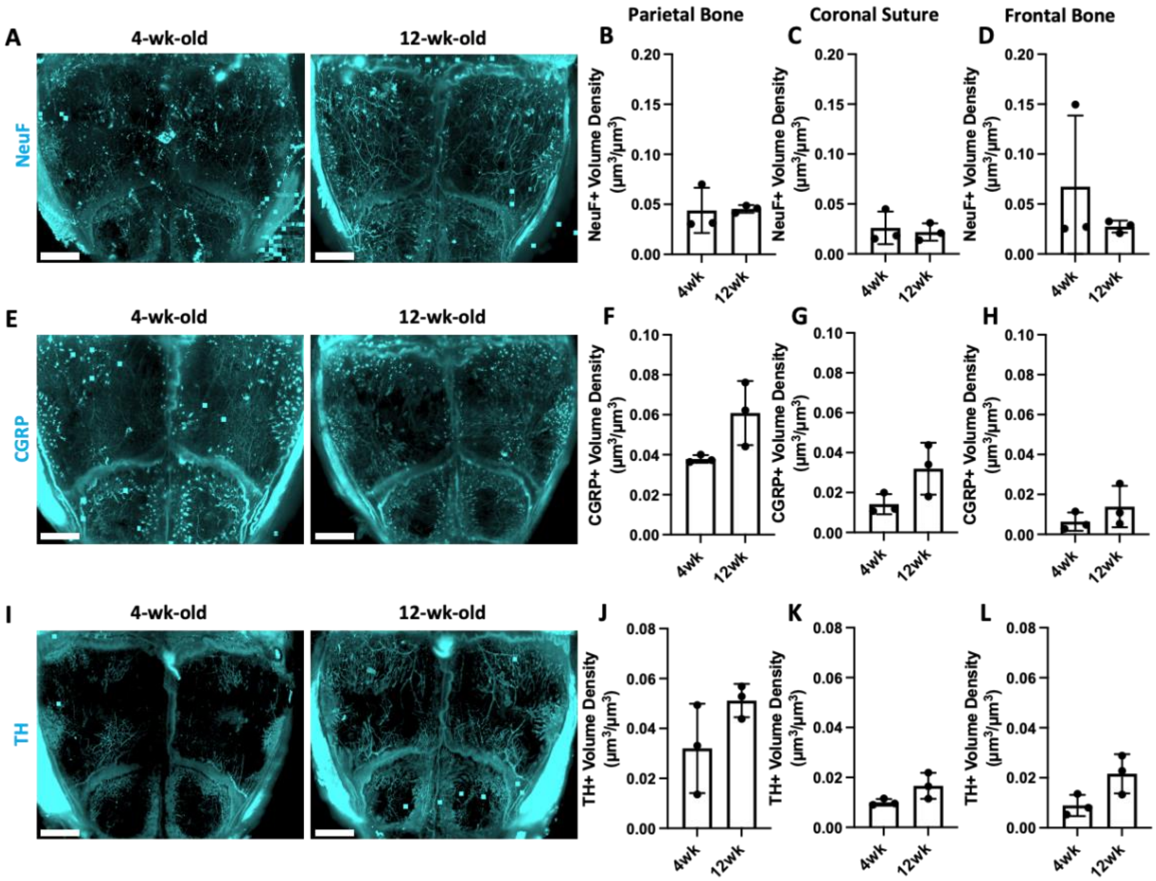

Supplemental Figure 7: Regional changes in nerve subtype distributions during postnatal development. A) Full calvaria MIP images of 4wk and 12wk mice with NeuF+ nerves (blue). Scale bar is 1500 µm. B-D) Nerve volume fraction calculations for 4wk and 12wk NeuF+ nerves in the B) Parietal Bone region, C) Coronal Suture region, and D) Frontal Bone region. D) Full calvaria maximum intensity projections of 4wk and 12wk mice with CGRP+ nerves (blue). Scale bar is 300 µm. F-H) Nerve volume fraction calculations for 4wk and 12wk CGRP+ nerves in the F) Parietal Bone region, G) Coronal Suture region, and H) Frontal Bone region. I) Full calvaria maximum intensity projections of 4wk and 12wk mice with TH+ nerves (blue). Scale bar is 300 µm. J-L) Nerve volume fraction calculations for 4wk and 12wk TH+ nerves in the J) Parietal Bone region, K) Coronal Suture region, and L) Frontal Bone region. Data are mean ± SD. Statistics were performed with a two-way ANOVA with post-hoc Tukey HSD test and a two-tailed t-test.

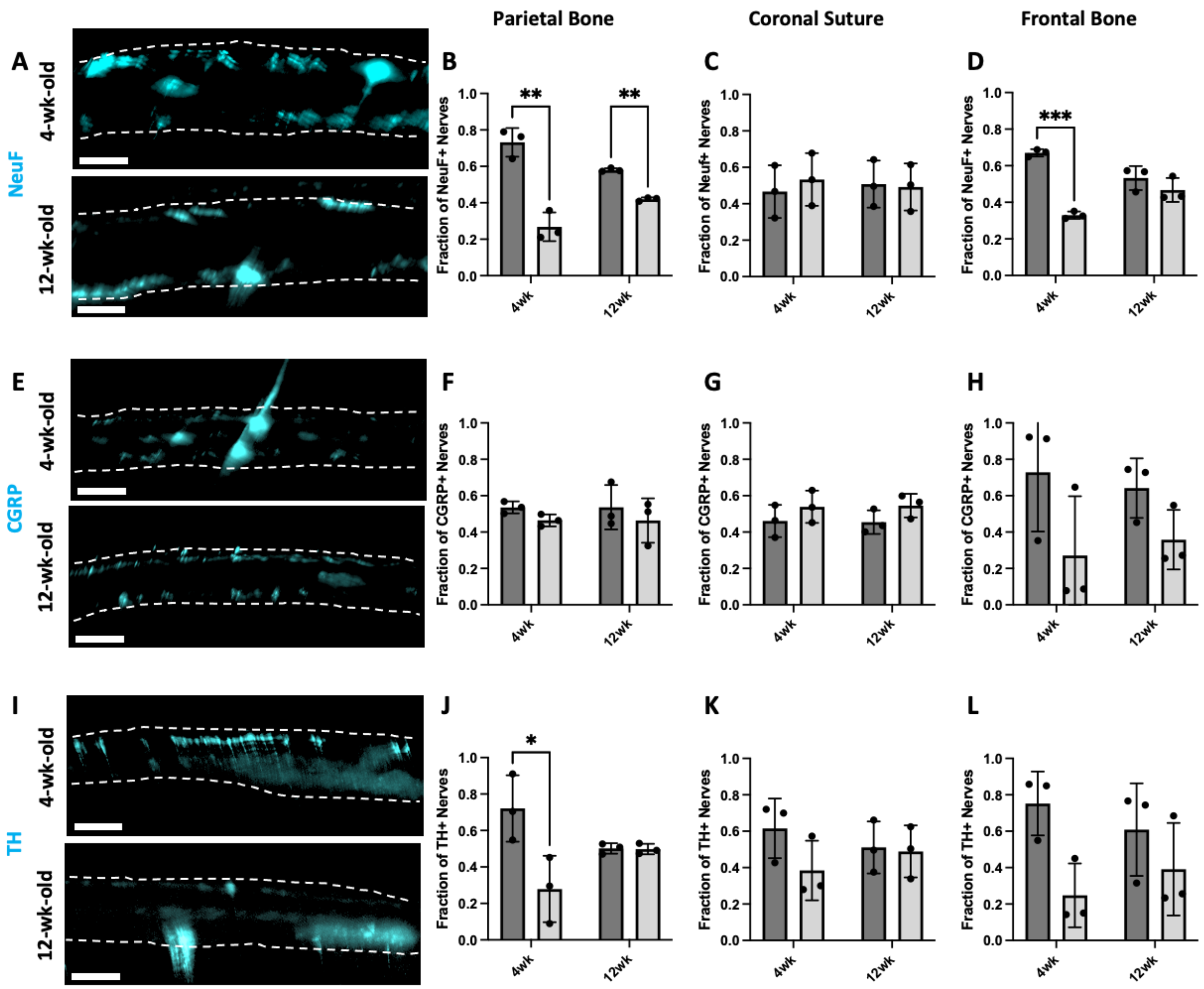

Supplemental Figure 8: Regional changes in nerve subtype dura and periosteum proportions during postnatal development. A) 50 µm coronal cross sections of NeuF+ nerves in 4wk and 12wk mice. Scale bar is 300 µm. B-D) Dura and periosteum calculations for 4wk and 12wk NeuF+ nerves in the B) Parietal Bone region, C) Coronal Suture region, and D) Frontal Bone region. D) 50 µm coronal cross sections of CGRP+ nerves in 4wk and 12wk mice. Scale bar is 300 µm. F-H) Dura and periosteum calculations for 4wk and 12wk CGRP+ nerves in the F) Parietal Bone region, G) Coronal Suture region, and H) Frontal Bone region. I) 50 µm coronal cross sections of TH+ nerves in 4wk and 12wk mice. Scale bar is 300 µm. J-L) Dura and periosteum calculations for 4wk and 12wk TH+ nerves in the J) Parietal Bone region, K) Coronal Suture region, and L) Frontal Bone region. Data are mean ± SD. Statistics were performed with a two-way ANOVA with post-hoc Tukey HSD test and a two-tailed t-test. *p<0.5 and ***p<0.01 where designated.

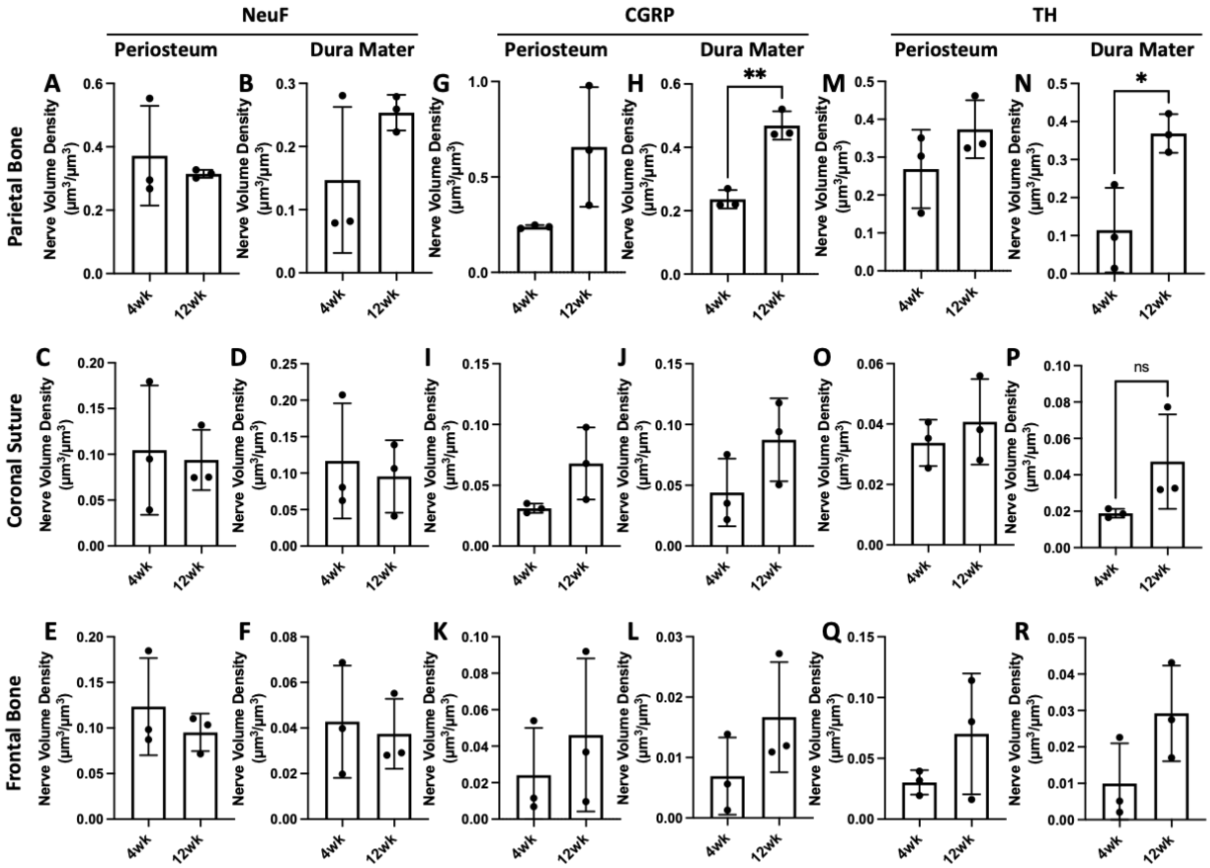

Supplemental Figure 9: Regional changes in nerve subtype dura and periosteum volume densities during postnatal development. A-F) Periosteal and dural nerve volume density calculations for NeuF+ nerves in 4wk and 12wk samples in A) Parietal Bone Region Periosteum, B) Parietal Bone Region Dura Mater, C) Coronal Suture Region Periosteum, D) Coronal Suture Region Dura Mater, E) Frontal Bone Region Periosteum, F) Frontal Bone Region Dura Mater. G-L) Periosteal and dural nerve volume density calculations for CGRP+ nerves in 4wk and 12wk samples in G) Parietal Bone Region Periosteum, H) Parietal Bone Region Dura Mater, I) Coronal Suture Region Periosteum, J) Coronal Suture Region Dura Mater, K) Frontal Bone Region Periosteum, L) Frontal Bone Region Dura Mater. M-R) Periosteal and dural nerve volume density calculations for TH+ nerves in 4wk and 12wk samples in M) Parietal Bone Region Periosteum, N) Parietal Bone Region Dura Mater, O) Coronal Suture Region Periosteum, P) Coronal Suture Region Dura Mater, Q) Frontal Bone Region Periosteum, R) Frontal Bone Region Dura Mater. Data are mean ± SD. Statistics were performed with a two-way ANOVA with post-hoc Tukey HSD test. *$p<0.5$ where designated.

Supplemental Table 1. List of all key reagents and resources used in this study.

Reagent or Resource	Source	Identifier
Antibodies		
Rabbit anti-mouse TUBB3 (1:200)	Abcam	ab18207
Rabbit anti-mouse NeuF (1:200)	Thermo Fisher Scientific	PA3-16721
Rabbit anti-mouse CGRP (1:200)	Sigma Aldrich	C8198
Rabbit anti-mouse TH (1:200)	Sigma Aldrich	AB152
Goat anti-mouse/rat CD31 (1:200)	R&D Systems	AF3628
Rat anti-mouse/rat Endomucin (1:50)	Santa Cruz Biotechnology	sc-65495
Donkey anti-goat AF800 plus, 0.67 mg/mL (1:100)	Thermo Fisher Scientific	A32930
Donkey anti-rabbit AF647 plus, 0.67 mg/mL (1:300)	Thermo Fisher Scientific	A32795
Donkey anti-rat biotin, 0.75 mg/mL (1:200)	Thermo Fisher Scientific	A18749
Streptavidin AF555 conjugate, 0.67 mg/mL (1:200)	Thermo Fisher Scientific	S32355
Reagents		
Heparin sodium salt from porcine mucosa	Sigma Aldrich	H3393-50KU
Paraformaldehyde, 16% aq. soln., methanol free	Alfa Aesar	433689M
Normal donkey serum	Sigma Aldrich	D9663-10ML
Trizma base	Sigma Aldrich	T6066-1KG
Trizma hydrochloride	Sigma Aldrich	T5941-1KG
Sodium chloride	Sigma Aldrich	S5886
Tween 20	Sigma Aldrich	P7949
Dimethylsulfoxide	Thermo Fisher Scientific	PI20688

Supplemental Methods

Confocal Imaging

For confocal imaging, we used a Zeiss LSM confocal equipped with 405, 488, 555, and 633 nm lasers. Following light sheet imaging, calvaria were mounted to a cover glass lined sample chamber and immersed in 100% TDE. Tile acquisitions were obtained of the parietal bone region. Tiles were overlapped by 15% to facilitate stitching. Both 10x and 20x lenses were used with a 2.5 µm step size. Laser intensities were set for each antibody and held consistent for each scan.

Calvaria Cryosectioning, Immunohistochemistry, and Cross-Section Imaging

Following all whole mount imaging, calvaria were embedded in Tissue-Tek O.C.T. Compound and flash frozen in liquid nitrogen. Calvaria were sectioned along the coronal direction on a cryostat (Leica CM3050 S) at a thickness of 30 µm. Sections were primarily collected from the parietal bone region. For immunohistochemistry, slides were blocked and permeabilized in 10% normal donkey serum with 0.1% Triton-X in PBS for 1 hour at RT. Next, slides were stained with TUBB3, CD31, and Emcn primary antibodies in 10% normal donkey serum in TBS-Tw at RT. After three washes for five minutes each in PBS, samples were stained with fluorophore conjugated secondary antibodies and DAPI for 1 hour in 10% normal donkey serum in TBS-Tw at RT. After three additional washes in PBS, slides were mounted with cover slips in a solution of 50% glycerol. Tiled z-stack images were taken with the 10x lens on a Zeiss AxioObserver with Apotome microscope.
